# Four Non-functional *FUT1* Alleles Were Identified in Seven Chinese Individuals with Para-Bombay Phenotypes

**Published:** 2018-08

**Authors:** Wei LIANG, Feng CAI, Liang YANG, Zhe ZHANG, Zhicheng WANG

**Affiliations:** 1. Dept. of Laboratory Medicine, Huashan Hospital, Shanghai Medical College, Fudan University, Shanghai, China; 2. Dept. of Laboratory Medicine, The Second People’s Hospital of Lianyungang City, Lianyungang, Jiangsu Province, China; 3. Dept. of Laboratory Medicine, Shanghai Municipal Hospital of Traditional Chinese Medicine, Shanghai University of Traditional Chinese Medicine, Shanghai, China; 4. Laboratory Department of Center Blood Station Ningbo, Ningbo, Zhejiang Province, China; 5. Health Bureau of Ningbo City, Ningbo, Zhejiang Province, China

**Keywords:** *FUT1*, *FUT2*, Para-Bombay phenotype

## Abstract

**Background:**

The para-Bombay phenotype is characterized by a lack of ABH antigens on red cells, but ABH substances are found in saliva. Molecular genetic analysis was performed for seven Chinese individuals serologically typed as para-Bombay in Blood Station Center of Ningbo, Zhejiang Province, Ningbo, China from 2011 to 2014.

**Methods:**

RBCs’ phenotype was characterized by standard serologic technique. Genomic DNA was sequenced with primers that amplified the coding sequence of α (1, 2)-fucosyltransferase genes *FUT1* (or *H*) and *FUT2* (or *Se*), respectively. Routine ABO genotyping analysis was performed. Haplotypes of *FUT1* were identified by TOPO cloning sequencing. Phylogenetic tree of H proteins of different organisms was performed using Mega 6 software.

**Results:**

Seven independent individuals were demonstrated to possess the para-Bombay phenotype. RBC ABO genotypes correlated with ABH substances in their saliva. *FUT1* 547delAG (*h1*), *FUT1* 880delTT (*h2*)*, FUT1 658T (h3)* and *FUT1* 896C were identified in this study. *FUT1* 896C was first revealed by our team. The *H*-deficient allele reported here was rare and the molecular basis for *H* deficient alleles was diverse as well in the Chinese population. In addition, the *FUT2* was also analyzed, only one *FUT2* allele was detected in our study: Se^357^. Phylogenetic tree of the H proteins showed that H proteins could work as an evolutionary and genetic marker to differentiate organisms in the world.

**Conclusion:**

Molecular genetic backgrounds of seven Chinese individuals were summarized sporadic and random mutations in the *FUT1* gene are responsible for the inactivation of the *FUT1*-encoded enzyme activity.

## Introduction

The Bombay and para-Bombay phenotypes are characterized by the deficiency of H, A, and B blood group antigens on the red blood cell (RBC) (1). The ABO locus on 9q34 determines the A and B antigens, while, α (1, 2)-fucosyltransferase genes *FUT1* and *FUT2* encode the H antigen, the precursor of A and B antigens. Both *FUT1* and *FUT2* gene encode α (1, 2)-fucosyltransferase and are closely linked on 19q13, showing 70% DNA sequence homology (2, 3), however, the biological role of them is distinct (4, 5), *FUT1* is the *H* gene expressed mainly on the membrane of the human erythrocytes and *FUT-2* is the *Se* gene expressed exclusively in the secretory glands and the digestive mucosa.

The para-Bombay phenotype is characterized by a non-functional *FUT1* gene accompanied by an active *FUT2* gene. The first mutant *FUT1* gene was identified in an India individual who lacked the H enzyme and had no H antigens on erythrocytes, which was a typical Bombay phenotype. To date, more than 43 silencing or weakening mutations have been described for FUT1 in the Blood Group Antigen Gene Mutation Database of the US National Center or Biotechnology Information. *FUT1* gene determines the synthesis of H type 1 (following A/B antigens) adsorbed onto the membrane of RBC from the plasma, but the encoded enzyme activity by a deficient *FUT1* gene is greatly abated, resulting in a lower amounts of H antigen (and A/B antigen) on the surface of RBC. In above situation, no matter the function of *FUT2* gene is normal or not, H antigen (and A/B antigen) is poorly expressed and can only be detected by adsorption-elution tests using proper the anti-H (and anti-A/B) reagents. The anti-H made from para-Bombay individuals usually shows a weaker reaction in the adsorption-elution test compared with the anti-H from individuals with the Bombay phenotype, which usually shows strong reactive with a wide thermal range, whereas, it is less reactive and even does not react above room temperature for anti-H from para-Bombay individuals. This paper described the molecular genetic backgrounds of seven such Chinese individuals.

## Materials and Methods

### Blood samples and saliva samples

Six probands with the para-Bombay phenotypes were identified during pre-transfusion testing in the time-period 2011 to 2014. One proband was a volunteer donor at the Ningbo Blood Station of Zhejiang Province in China, whose erythrocytes showed the rare phenotype with a cell and serum grouping discrepancy was suspected to be a para-Bombay individual. Overall, 5 mL of peripheral blood was bled with ethylenediaminetetraacetic acid dipotassium (EDTA-2K) anticoagulant from each individual. Saliva samples were presented by all the suspected para-Bombay individuals as well. ABH antigens on erythrocytes and in saliva were examined as well.

Genomic DNA was extracted from whole blood samples using a DNA isolation kit (Qiagen, Hilden, Germany) according to the manufacturer’s instruction. The DNA of peripheral blood from 110 randomly chosen Chinese individuals with normal ABO blood group phenotypes were isolated to assess the frequency of *H* allele in natural population. All the subjects signed the informed consents

### Blood group serological studies

ABO serology was performed with standard serological techniques. The adsorption-elution test (6) was used to detect trace amounts of A/B antigens on red blood cells or H antigens in sera. The haemagglutination inhibition test was employed to detect whether ABH substances were present or not in saliva (6). Lewis blood group was also tested to know the secretory type. For routine testing, one drop of anti-A, -B (Shenxing, Shanghai, China), anti-H, anti-AB, anti-Le^a^ and anti-Le^b^ (Sanquin, Amsterdam, Netherlands) was placed in a tube and mixed with washed RBCs, respectively. After centrifugation, the results of haemagglutination were observed macroscopically and microscopically. The human anti-A, B was prepared by our laboratory.

### ABO genotyping

The *ABO* preliminary genotypes were determined using a Sequence-specific-primer–PCR (PCR-SSP) technology designed by our team with Primer Premier 5.0 (Premier, Palo Alto, CA). All primers were synthesized by Life Technologies (Invitrogen, Life Technologies, USA). The sequences are given in Table 1.

**Table 1:** Primers and PCR conditions used in the analysis of *ABO*, *FUT1* and *FUT2* genes

	***Primer name***	***Sequence(5/ to 3/)***	***Annealing Temperature***	***PCR Product(bp)***
*ABO* gene	SSP 261F	GCTTGCTGTGTGTTCCCGCAGGTCC		
	SSP 261GR	AATGGGAGCCAGCCAAGGGGTCA	70 °C	280
	SSP 261AR	CAATGGGAGCAAGCCAAGGAGTA	64 °C	279
	SSP 703F	TGCTGCTCTAAGCCTTCCAATG		
	SSP 703AR	CGGCTGCTTCCGTAGAAGAT	60 °C	460
	SSP 703GR	CGGCTGCTTCCGTAGAATCC	62 °C	460
	ABO E6F	TGGTCAGAGGAGGCAGAA		
	ABO E6R	CTCAATGTCCACAGTCACTC	62 °C	316
	ABO E71F	TGCTGCTCTAAGCCTTCCAATG		
	ABO E71R	TGCCGAACAGCGGAGTCAG	64 °C	429
	ABO E72F	GGTGGATTACCTGGTGTGCGTG		
	ABOE72R	AAACAGAGTTTACCCGTTCTGCT	62 °C	450
*FUT1* gene	FUT1-1F	CTCCCTTACCCCACATCCCT		
	FUT1-1R	CTGAGGCATAACCTGCAGATAGT	66 °C	771
	FUT1-2F	TTCACGACTGGATGTCGGAG		
	FUT1-2R	CTAGAAAGATCAGGCTACTTC	62 °C	701
*FUT2* gene	FUT2F	CCATCTCCCAGCTAACGTGTCC		
	FUT2R	GGGAGGCAGAGAAGGAGAAAAGG	64 °C	1118

F: Forward primer, R: reverse primer, GR, AR: The reverse primer specified for an allele of ABO gene, whose certain site is base G, A, respectively

### Sequencing of ABO exons 6 and 7

ABO exact genotypes were determined by sequencing of exons 6 and 7of *ABO* gene, whose primers used, are listed in Table 1. DNA fragments were amplified with primers ABO-E6F and ABO-E6R for exon 6 or primers ABO-E71F, ABO-E71R, ABO-E72F and ABO-E72R for exon 7. In order to acquire clearer sequence diagrams, two pairs of primers were designed for exon 7. The 50μL reaction mixture contained 25μL 2×dNTP (TIANGEN, Beijing, China), 2.5 μL of each primer (Invitrogen, Life Technologies, USA), 200 ng of genomic DNA and water. After initial denaturation at 95 °C for 1 min, the reaction mixtures were subjected to 35 cycles of denaturation at 95 °C for 25 sec, followed by annealing at each optimal temperature (Table 1) for 25 sec and extending at 75 °C for 45 sec, plus a final extension at 72 °C for 5 min. PCR products were separated on a 1.5% agarose gel (Biowest, Gene Company, Spain), all showed a single bright band, then the PCR products were purified and unidirectionally sequenced with an ABI BigDye Terminator Cycle Sequencing kit (Applied Biosystems, CA, USA) and Universal DNA Purification Kit (TIANGEN, Beijing, China) according to the manufacturer’s instructions, respectively. The sequence data were analyzed by FinchTV1.4 software (Geospiza, Seattle, USA) and the *ABO* genotypes were assigned according to the nucleotides at the polymorphic *ABO* positions. All the acquired nucleotides sequences were compared with standard *ABO* polymorphisms from the dbRBC of NCBI and each SNP or mutation was analyzed and documented in the *ABO* gene.

### Sequencing of FUT1

Two DNA fragments covering the entire coding region (1098bp) were amplified to identify the mutations in the *FUT1*. The reagents and protocols used in the PCR were the same as the sequencing of *ABO gene* mentioned in the above section. The sequence data were analyzed by FinchTV1.4 software (Geospiza, Seattle, USA) and all achieved nucleotides sequences were compared with standard *Hh* polymorphisms from the dbRBC of NCBI, and every mutation in the *FUT1* gene was analyzed, each *FUT1* genotype was assigned at last.

### Analysis of FUT1 haplotype

In order to analyze the haplotype, the PCR-product of *FUT1* gene was ligated into the plasmid pCRIITOPO, then the competent cells of TOP-10 *Escherichia coli* were transfected with the recombinant plasmids using a TOPO TA cloning kit (Invitrogen, Carlsbad, CA, USA) according to the manufacturer’s instructions. The colonies on LB plates were selected randomly and screened using colony-PCR for each sample. Plasmid DNA of positive colony was extracted by a kit (TIANGEN Beijing, China) and used as templates for the sequencing reaction. The PCR products were sent to Shanghai Sunny Biotechnology Co., Ltd (Sunny, Shanghai, China), where all the following experiment steps were finished.

### Sequencing of FUT2

To analyse the genotyp*e of FUT2*, the whole coding region (1118bp) of *FUT2* was amplified using the primers (Table 1). The primer design and PCR amplification of *FUT2* were performed (7).

### Phylogenetic analysis

**Fig. 1:**
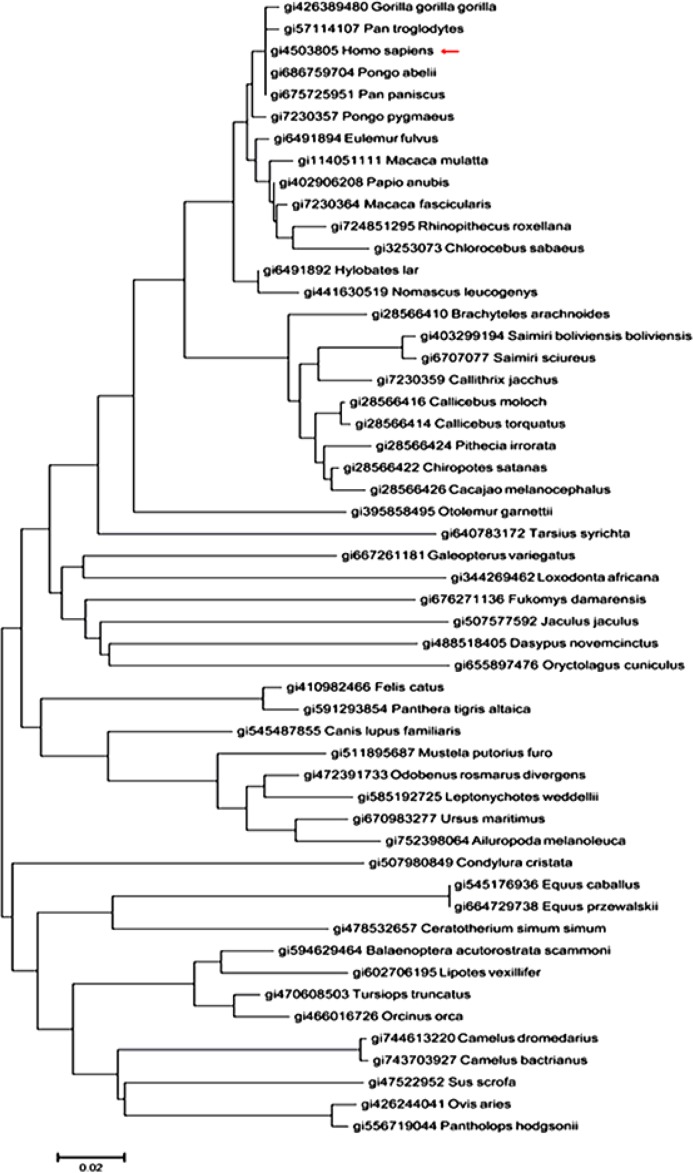
Evolutionary relationships of taxa The red arrow points at the branch of Human sapiens, phylogenetic tree shows that the Gorilla, Pan troglodytes, Pongo abelii, and paniscus are closer in the evolution distance comparing to other organisms

Human sapiens H protein (gi4503805) sequence as query sequence was pasted in the text area of BlastP, and 52 organisms who express the H proteins were searched out, every protein sequence was downloaded in FASTA format. The evolutionary history (Fig. 1) was inferred using the Neighbor-Joining method (8).

The evolutionary distances were computed using the Poisson correction method (9) and the evolutionary analyses were conducted in MEGA6 (10).

## Results

### Serological results and ABO genotypes

The ABH substances on RBCs could not be detected using direct agglutination, even all the reagents, polyclonal, monoclonal anti-sera and the lectin Ulex europaeus (anti-H) were chosen to perform such experiment (Table 2). However, the microscale A and/or B antigens on red cells were detected by the absorption-elution assay. The presence of ABH substances in saliva was consistent with their Le (a–b+) phenotypes.

**Table 2:** Phenotypes and genotypes of 7 Chinese para-Bombay individuals

***No***	***Haemagglutination***				***Absorption-elution***		***Antigens in saliva***		***Anti-H In serum***	***Genotypes***			***Para-Bombay Phenotype***
	***A***	***B***	***H***	***Lewis***	***A***	***B***	***A***	***B***		***ABO***	***FUT1***	***FUT2***	
1	−	−	−	a−b+	−	+	−	+	+	B101/O01	h1/h1	Se^357^/Se^357^	B
2	−	−	−	a−b+	+	−	+	−	+	A102/O02	h1/h3	Se^357^/Se^357^	A
3	−	−	−	a−b+	+	+	+	+	+	A102/B101	h1/h1	Se^357^/Se^357^	AB
4	−	−	−	a−b+	+	−	+	−	+	A101/O01	h1/h3	Se^357^/Se^357^	A
5	−	−	−	a−b+	+	−	+	−	+	A102/O02	h2/h2	Se^357^/Se^357^	A
6	−	−	−	a−b+	+	−	+	−	+	A102/O01	h1/h3	Se^357^/Se^357^	A
7	−	−	−	a−b+	−	+	−	+	+	B101/O02	h1/h?	Se^357^/Se^357^	B

−: absent; +: present; *ABO* phenotypes were determined by adsorption and elution tests; h?: denotes *FUT1* 896C

### The analysis of the FUT1 gene

Three different mutations (*h1*, *h2* and *h3*) were detected in the six individuals with the para-Bombay phenotypes using DNA sequencing based on the entire *FUT1* coding region. The genotypes of heterozygous (*h1h3*) or homozygous (*h1h1*, *h2h2*) were identified (Table 2), according to the nomenclature for non-functional *FUT1* alleles (11). However, for the case 7, two heterozygous mutations of the *FUT1*, 547-552AGAGAG/AGAG, and 896T/C were identified by our team (12). Analysis of sequences homologous to human *FUT1* showed that Gln299 was conserved in the *FUT1* enzymes of 16 other mammals reported to date (Table 3), which suggested that Gln299 of the human *FUT1* enzyme may be important in maintaining the biological function.

**Table 3:** Amino acid sequence alignment for *FUT1* enzyme

***Species***	***Accession no***	***Amino acid sequence alignment***																					
Homo Sapiens	NP_000139	291	W	K	D	F	A	L	L	T	Q	C	N	H	T	I	M	T	I	G	T	F	310
Gorilla	AAF14067	292	-	-	-	-	-	-	-	-	-	-	-	-	-	-	-	-	-	-	-	-	311
*Pan troglodytes*	AAF14065	292	-	-	-	-	-	-	-	-	-	-	-	-	-	-	-	-	-	-	-	-	311
*Pongo pygmaeus*	AAF42964	278	-	-	-	-	-	-	-	-	-	-	-	-	-	-	-	-	-	-	-	-	297
*Macaca fascicularis*	AAF42967	292	-	-	-	-	-	-	-	-	-	-	-	-	-	-	-	-	-	-	-	-	311
*Macaca mulatta*	AAF14069	292	-	-	-	-	-	-	-	-	-	-	-	-	-	-	-	-	-	-	-	-	311
*Chlorocebus sabaeus*	BAA29047	292	-	-	-	-	-	-	-	-	-	-	-	-	-	-	-	-	-	-	-	-	311
*Hylobates lar*	AAF14062	291	-	-	-	-	-	-	-	-	-	-	-	-	-	-	-	-	-	-	-	-	310
*Eulemur fulvus*	AAF14063	292	-	-	-	-	-	-	-	-	-	-	-	-	-	-	-	-	-	-	-	-	311
*Saimiri sciureus*	AAF25584	292	-	-	-	-	-	-	-	A	-	-	-	-	-	-	-	-	-	-	-	-	311
*Callithrix jacchus*	AAF42965	291	-	-	-	-	-	-	-	-	-	-	-	-	-	-	-	-	-	-	-	-	310
*Sus scrofa*	AAB02984	291	A	R	-	-	-	-	-	V	-	-	-	-	-	-	-	-	-	-	-	-	310
*Oryctolagus cuniculus*	Q10979	291	A	-	-	-	-	-	-	-	-	-	-	-	-	V	-	-	-	-	-	-	310
*Bos taurus*	AAF07933	291	N	-	-	-	-	-	-	-	-	-	-	-	-	-	-	-	-	-	-	-	310
*Mus musculus*	AAF45145	293	G	-	-	-	-	-	-	-	-	-	-	-	-	-	-	-	-	-	-	-	312
*Mus spicilegus*	BAB68637	293	G	-	-	-	-	-	-	-	-	-	-	-	-	-	-	-	-	-	-	-	312
*Rattus norvegicus*	NP_112515	292	G	-	-	-	-	-	-	-	-	-	-	-	-	-	-	-	-	-	-	-	311

Dashes symbolize amino acid sequences identity with the human sequence. The affected amino acid in the *FUT1* 896C allele is underlined

### The analysis of the FUT2 gene

The relevant ABH antigens were detected in the saliva for each individual, which showed that an active *FUT2* gene existed in each individual. The homozygous mutation 357T was observed in each individual by direct DNA sequencing compared with the reference sequence (GenBank accession no. U17894) in the coding region. The 357C>T variant of *FUT2* did not result in an amino acid change, are common in Asian populations (13).

### Phylogenetic analysis

Phylogenetic tree was portrayed, showing that H proteins could work as an evolutionary and genetic marker to differentiate organisms in the world.

## Discussion

In the present study, we detected seven individuals; all of them possessed the para-Bombay phenotype, having the distinct genetic background, respectively. Four non-functional *FUT1* alleles were tested by DNA sequencing based on the entire *FUT1* coding region, including three reported defective *FUT1* alleles: *FUT1* 547delAG (*h1*), *FUT1* 880delTT (*h2*), *FUT1* 658T (*h3*) and a novel *FUT1* allele, *FUT1* 896C (13). Both alleles’ *h1* and *h2* are two-base deletions: the AG deletion is located at nucleotides 547–552 for *h1* and the TT bases are deleted at nucleotides 880–882 for *h2*. The *h3* allele contains a C658 to T missense mutation, which results in a change from Arg to Cys at amino acid position 220. These mutations were also reported in individuals with the para-Bombay phenotypes in other places (11,14). *FUT1* 896 C was first revealed by our team. The H-deficient allele reported here was, as expected, rare in the Chinese population and the molecular basis for H deficient alleles was diverse as well. In addition to the *FUT1*, the *FUT2* was also analyzed, only one *FUT2* allele was detected in our study: Se^357^. Se^357^ allele was very common in the Asian populations (7, 14–16). *FUT2* gene analysis results were consistent with the subjects’ secretor status. Different ethnic and/or geographic mutations are revealed for the *FUT2* gene and some of the mutations could result in a non-secretor phenotype. The prevalent synonymous mutation for *FUT2* gene is 357C>T in Asian populations compared with counterpart, the nonsense mutation 428G>A in the African and Caucasian populations (17). “The relatively high allele frequency for some of the *FUT2*-null alleles is likely an evolutionary advantage when the soluble and/or mucosal H antigens are absent, and the presence of H determinants on mucosal surfaces may be more biologically important than their cellular analogs, various reports of the increased resistance to infection by a wide range of pathogens in individuals of the nonsecretor phenotype supported the observations (18–21).”

The occurrence rates of *FUT1* mutations, resulting in Bombay and para-Bombay phenotypes vary from an estimated 1:1,000,000 in Europe to 1:1000 on Reunion Island (13). In a large Caucasian population, the total frequency of nonfunctional alleles of the *FUT1* has been estimated to be as high as 1:347 (22). In the whole Japanese population, the incidence of Bombay and para-Bombay conjectured is approximately one in two or 300000 (23). There are more para-Bombay phenotypes than Bombay in the Chinese population. Data showed that the incidence of *FUT1* mutations were 1/8000–1/10000, 1–15620 in Taiwan and Hong Kong, respectively (7,11).

To date, more than 43 effective mutations have been documented for *FUT1.* The mutation, giving rise to Bombay phenotype was first described (24), *FUT1* 725T>G, together with the deletion of the *FUT2* gene has been detected only in subjects from subcontinental Indian (25). Another preferment mutation is the *FUT1* 349C>T, usually found on the island of Reunion, moreover, *FUT1* 547delAG(*h1*), *FUT1* 880delTT(*h2*), *FUT1* 658T(*h3*) mutation was found mainly in Chinese population (14, 16, 26). *FUT1* 695A, *FUT1* 990delG, *FUT1* 721C mutation was prevalent in Japanese (23) and so on. The mutation of *FUT1* gene is closely related to the geographical regions, demonstrated by this study.

In contrast, non-functional *FUT2* mutations are keeping at a relative steady frequency, about 20% in most populations. In European and African populations, the most prevalent nonsense mutation is 428G>A, with an allele frequency of 0.47 and 0.416, respectively (27). In Asian populations, the allele harboring both the synonymous mutation 357C>T and the inactivating mutation 385A>T is the main cause of the nonsecretor phenotype with a frequency of 0.406 (28, 29). The inactivation of the *FUT1* gene happened after *FUT2* gene inactivation, as all of the Bombay and nonsecretor para-Bombay individuals had the same inactivated *FUT2* allele but possessed distinct inactivated *FUT1* alleles (23), according to our study, there might be some specific selective advantage on the individuals with the mutant *FUT2* alleles, but some selective disadvantage on the individuals with the mutant *FUT1* alleles. *FUT2* mutations were more ethnically specific and may be used as anthropologic markers (27, 30).

## Conclusion

Four non-functional *FUT1* alleles (*h1*, *h2*, *h3*, *FUT1* 896C) were identified in seven Chinese individuals with para-Bombay phenotypes and on the same Se^357^/Se^357^ haplotype background. As the para-Bombay phenotype is rare in the natural population, it may bring troubles in clinical blood transfusion, blood typing and so on; this article would contribute to understanding the special blood group not only in theory but also in practice.

## Ethical considerations

Ethical issues (Including plagiarism, Informed consent, misconduct, data fabrication and/or falsification, double publication and/or submission, redundancy, etc.) have been completely observed by the authors.

## References

[1] Zhang A Chi Q Ren B (2015). Genomic analysis of para- Bombay individuals in southeastern China: the possibility of linkage and disequilibrium between FUT1 and FUT2. Blood Transfus, 13(3): 472–477. 25761312 10.2450/2015.0185-14 PMC4614301

[2] Ball SP Tongue N Gibaud A (1991). The human chromosome 19 linkage group FUT1 (H), FUT2 (SE), LE, LU, PEPD, C3, APOC2, D19S7 and D19S9. Ann Hum Genet, 55(Pt 3): 225–233. 1763885 10.1111/j.1469-1809.1991.tb00417.x

[3] Reguigne-Arnould I Couillin P Mollicone R (1995). Relative positions of two clusters of human alpha-L-fucosyltransferases in 19q (FUT1-FUT2) and 19p (FUT6-FUT3-FUT5) within the microsatellite genetic map of chromosome 19. Cytogenet Cell Genet, 71(2): 158–162. 7656588 10.1159/000134098

[4] Oriol R Danilovs J Hawkins BR (1981). A new genetic model proposing that the Se gene is a structural gene closely linked to the H gene. Am J Hum Genet, 33(3): 421–431. 7246545 PMC1685044

[5] Le Pendu J Cartron JP Lemieux RU (1985). The presence of at least two different H-blood-group-related beta-D-gal alpha-2-L-fucosyltrans- ferases in human serum and the genetics of blood group H substances. Am J Hum Genet, 37(4): 749–760. 9556663 PMC1684624

[6] Matsushita M Otani K Sakamoto Y (2015). Increase in Alkaline Phosphatase Activity after High-Fat Meal Ingestion is Correlated to the Amount of ABH Substances in Saliva. Rinsho Byori, 63(5): 543–547. 26524892

[7] Yip SP Chee KY Chan PY (2002). Molecular genetic analysis of para-Bombay phenotypes in Chinese: a novel non-functional FUT1 allele is identified. Vox Sang, 83(3): 258–262. 12366770 10.1046/j.1423-0410.2002.00184.x

[8] Saitou N Nei M (1987). The neighbor-joining method: a new method for reconstructing phylogenetic trees. Mol Biol Evol, 4(4): 406–425. 3447015 10.1093/oxfordjournals.molbev.a040454

[9] Zuckerkandl E Pauling L (1965). Evolutionary divergence and convergence in proteins. Edited in Evolving Genes and Proteins by Bryson V Vogel HJ, Academic Press New York, 97–166.

[10] Tamura K Stecher G Peterson D (2013). MEGA6: Molecular Evolutionary Genetics Analysis version 6.0. Mol Biol Evol, 30(12): 2725–9. 24132122 10.1093/molbev/mst197 PMC3840312

[11] Yu LC Yang YH Broadberry RE (1997). Heterogeneity of the human H blood group alpha(1,2)fucosyltransferase gene among para-Bombay individuals. Vox Sang, 72(1): 36–40. 9031499 10.1046/j.1423-0410.1997.00036.x

[12] Liang W Xu H Liu YY (2015). Molecular genetic analysis of para-Bombay phenotype in Chinese persons: a novel FUT1 allele is identified. Transfusion, 55(6 Pt 2): 1588. 25858679 10.1111/trf.13022

[13] Blumenfeld OO Patnaik SK (2004). Allelic genes of blood group antigens: a source of human mutations and cSNPs documented in the Blood Group Antigen Gene Mutation Database. Hum Mutat, 23(1): 8–16. 14695527 10.1002/humu.10296

[14] Cai XH Jin S Liu X (2011). Molecular genetic analysis for the para-Bombay blood group revealing two novel alleles in the FUT1 gene. Blood Transfus, 9(4): 466–468. 21839020 10.2450/2011.0115-10 PMC3200418

[15] Wang B Koda Y Soejima M (1997). Two missense mutations of H type alpha(1,2)fucosyltransferase gene (FUT1) responsible for para-Bombay phenotype. Vox Sang, 72(1): 31–35. 9031498 10.1046/j.1423-0410.1997.00031.x

[16] Xu X Tao S Ying Y (2011). A novel FUT1 allele was identified in a Chinese individual with para-Bombay phenotype. Transfus Med, 21(6): 385–393. 21988368 10.1111/j.1365-3148.2011.01111.x

[17] Kelly RJ Rouquier S Giorgi D (1995). Sequence and expression of a candidate for the human Secretor blood group alpha(1,2)fucosyltransferase gene (FUT2). Homozygosity for an enzyme-inactivating nonsense mutation commonly correlates with the non-secretor phenotype. J Biol Chem, 270(9): 4640–9. 7876235 10.1074/jbc.270.9.4640

[18] Storry JR Johannesson JS Poole J (2006). Identification of six new alleles at the FUT1 and FUT2 loci in ethnically diverse individuals with Bombay and Para-Bombay phenotypes. Transfusion, 46(12): 2149–2155. 17176328 10.1111/j.1537-2995.2006.01045.x

[19] Ali S Niang MA N’doye I (2000). Secretor polymorphism and human immunodeficiency virus infection in Senegalese women. J Infect Dis, 181(2): 737–739. 10669366 10.1086/315234

[20] Thorven M Grahn A Hedlund KO (2005). A homozygous nonsense mutation (428G-->A) in the human secretor (FUT2) gene provides resistance to symptomatic norovirus (GGII) infections. J Virol, 79(24): 15351–5. 16306606 10.1128/JVI.79.24.15351-15355.2005 PMC1315998

[21] Kindberg E Hejdeman B Bratt G (2006). A nonsense mutation (428G-->A) in the fucosyltransferase FUT2 gene affects the progression of HIV-1 infection. AIDS, 20(5): 685–9. 16514298 10.1097/01.aids.0000216368.23325.bc

[22] Wagner FF Flegel WA (1997). Polymorphism of the h allele and the population frequency of sporadic nonfunctional alleles. Transfusion, 37(3): 284–290. 9122901 10.1046/j.1537-2995.1997.37397240210.x

[23] Kaneko M Nishihara S Shinya N (1997). Wide variety of point mutations in the H gene of Bombay and para-Bombay individuals that inactivate H enzyme. Blood, 90(2): 839–849. 9226185

[24] Bhende YM Deshpande CK Bhatia HM (1994). A “new” blood-group character related to the ABO system. 1952. Indian J Med Res, 99: 3p. 8005636

[25] Koda Y Soejima M Johnson PH (1997). Missense mutation of FUT1 and deletion of FUT2 are responsible for Indian Bombay phenotype of ABO blood group system. Biochem Biophys Res Commun, 238(1): 21–25. 9299444 10.1006/bbrc.1997.7232

[26] Yan L Zhu F Xu X (2005). Molecular basis for para-Bombay phenotypes in Chinese persons, including a novel nonfunctional FUT1 allele. Transfusion, 45(5): 725–730. 15847661 10.1111/j.1537-2995.2005.04305.x

[27] Liu Y Koda Y Soejima M (1998). Extensive polymorphism of the FUT2 gene in an African (Xhosa) population of South Africa. Hum Genet, 103(2): 204–210. 9760207 10.1007/s004390050808

[28] Henry S Mollicone R Fernandez P (1996). Molecular basis for erythrocyte Le(a+ b+) and salivary ABH partial-secretor phenotypes: expression of a FUT2 secretor allele with an A-->T mutation at nucleotide 385 correlates with reduced alpha(1,2) fucosyltransferase activity. Glycoconj J, 13(6): 985–993. 8981090 10.1007/BF01053194

[29] Kudo T Iwasaki H Nishihara S (1996). Molecular genetic analysis of the human Lewis histo-blood group system. II. Secretor gene inactivation by a novel single missense mutation A385T in Japanese nonsecretor individuals. J Biol Chem, 271(16): 9830–7. 8621666 10.1074/jbc.271.16.9830

[30] Koda Y Ishida T Tachida H (2003). DNA sequence variation of the human ABO-secretor locus (FUT2) in New Guinean populations: possible early human migration from Africa. Hum Genet, 113(6): 534–541. 14569463 10.1007/s00439-003-1013-6

